# Effects of altitude and exercise intensity on cardiac function in rats

**DOI:** 10.1113/EP092037

**Published:** 2024-09-26

**Authors:** Minxia Zhu, Xiaofeng Li, Bing Liu, Jing Guo, Yuanyuan Xiao, Zhiyao Liu, Mengru Duan, Yi Liu

**Affiliations:** ^1^ Key Laboratory of High Altitude Hypoxia Environment and Life Health, School of Medicine Xizang Minzu University Xianyang Shaanxi P. R. China; ^2^ Xizang Minzu University Xianyang Shaanxi P. R. China

**Keywords:** altitude, cardiac function, exercise, myocardial injury

## Abstract

High‐altitude exercise affects cardiac function. This study investigated how altitude and exercise intensity interacted to affect cardiac function of Sprague‐Dawley rats. Four altitudes (410, 3600, 4600 and 5600 m) and three exercise intensities (non‐exercise (N), low‐intensity exercise (L) and high‐intensity exercise (H)) were tested combinatorically. After 4 weeks of exercise, cardiac function and specific markers of myocardial injury were assessed. With regard to cardiac function, (a) at the same intensity, stroke volume and left ventricular end‐diastolic volume were higher in the 3600 m group but lower in the 4600 and 5600 m groups; and (b) the heart rate increased with altitude and intensity. The biochemical results showed that the levels of creatine kinase, myoglobin and cardiac troponin I generally increased with increasing altitude and exercise intensity, significantly for creatine kinase and myoglobin at 4600 and 5600 m. For pathological results, (a) in the non‐exercise group, pathological damage was observed only in the 5600 N group; and (b) in the exercised state, varying degrees of injury were noted, except for the 410 and 3600 L groups. There may be a turning point at 3600 m where the injury to the heart increases. Myocardial injury markers exhibited abnormalities before cardiac dysfunction. Detecting these markers is crucial to provide warnings for the individual from cardiac disease during high‐altitude exercise.

## Abstract (highlights)

- **What is the central question of this study?**
  Exercise at high altitude increases the burden of heart, thus affecting cardiac function and causing myocardial damage: what changes occur for markers of myocardial injury, cardiac function and structure, and what is the turning point of cardiac injury?
- **What is the main finding and its importance?**
  As the altitude and exercise intensity increase, there may be a turning point at 3600 m where the injury to the heart increases. Myocardial injury markers show abnormalities before cardiac dysfunction. It is important to detect these markers to provide a warning for the individual from cardiac disease while exercising at high altitude.

## 1 INTRODUCTION

Globally, over 500 million individuals inhabit high‐altitude regions (≥1500 m), accounting for approximately 6.58% of the world's population (Tremblay & Ainslie, 2021). The Qinghai–Tibetan Plateau is the highest plateau in the world and has been settled for generations, at present accommodating approximately 5 million inhabitants. Notably, more than 70% of this populace reside at altitude exceeding 3500 m (Chao‐ying et al., 2017). Exercise serves as a potent way to enhance both physical fitness and overall health (Qiu et al., 2023). Cardiac function not only reflects an individual's well‐being but also determines the intensity of exercise they can bear. While exercise can improve cardiac function, it simultaneously elevates myocardial oxygen consumption. At high altitudes, the prevalence of hypoxia exacerbates the cardiac workload, potentially compromising cardiac function and even precipitating myocardial damage (Cattadori et al., 2018; Izquierdo et al., 2021). Thus, careful consideration must be given to the balance between exercise benefits and potential myocardial injury in such environments.

The Qinghai–Tibetan Plateau stands as the world's most densely populated plateau, and this study adopts Tibet as an exemplary high‐altitude region to delve into the cumulative impacts of varying altitudes and exercise intensities on the heart, examining these effects through the integrating lens of myocardial enzyme secretion, cardiac structural alterations and functional performance. By pinpointing a threshold for exercise benefits and potential myocardial injury, this research offers invaluable insights for the tailored development of exercise recommendations in high‐altitude environments, thereby fostering advancements in fundamental research within the realm of high‐altitude sports science and medicine.

## 2 METHODS

### 2.1 Ethical approval

This study achieved ethical approval from the Ethics Committee at Medical College of Xizang Minzu University (No. 201906), and all experiments complied with the UK Animals (Scientific Procedures) Act 1986.

### 2.2 Animals

Ninety‐six adult male Sprague–Dawley (SD) rats, initial weight 180–220 g, 7–8 weeks old, were procured from the Laboratory Animal Center, Xi'an Jiaotong University (certification number: SCXK (SHAAN) 2018‐001) and allowed to acclimate for 1 week. The ambient temperature was kept at 25 ± 2°C, and the humidity was between 50% and 60%. The rats lived in a 12 h light–dark cycle every day with free access to food and water.

### 2.3 Exercise plan

**FIGURE 1:**
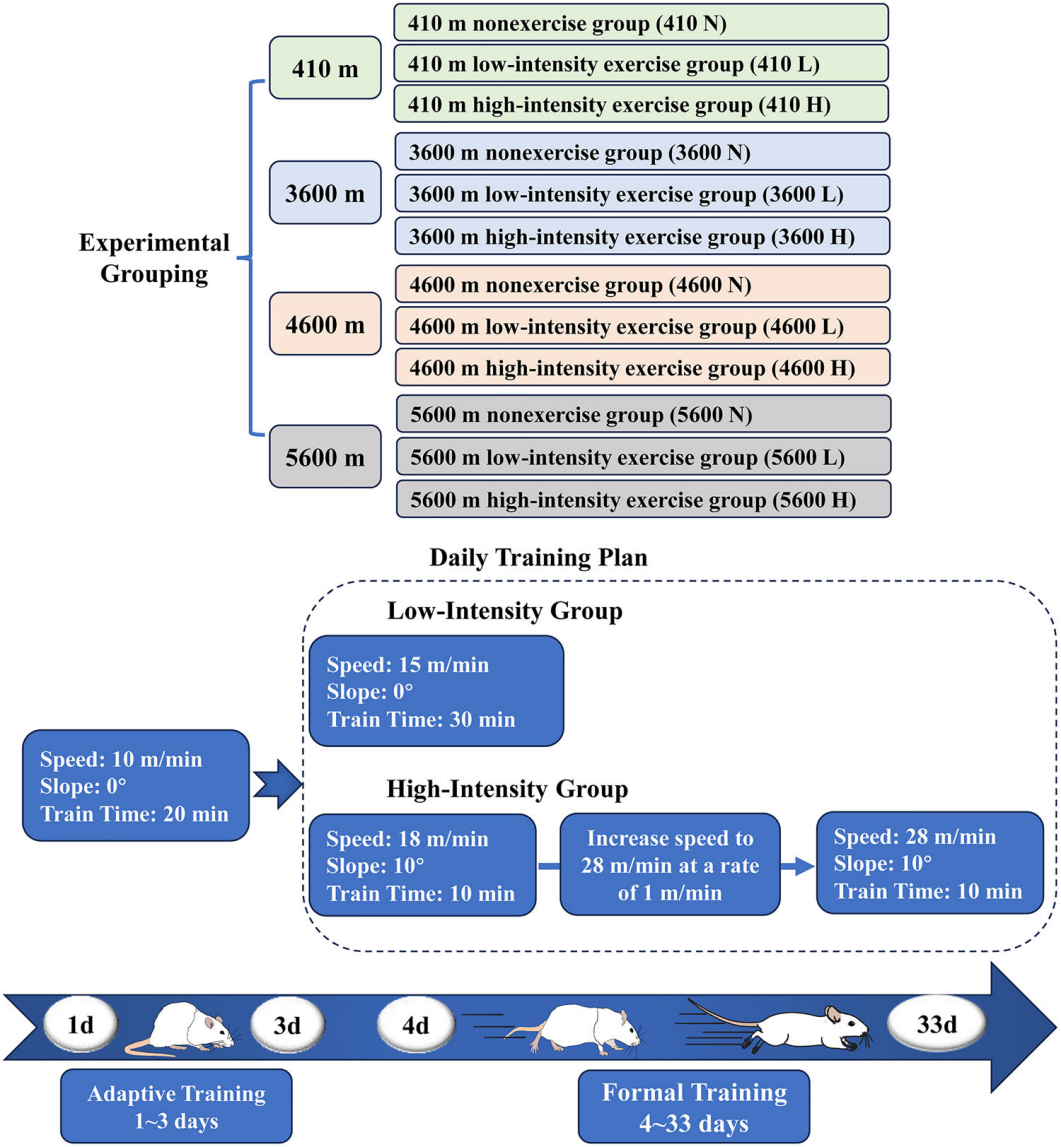
Experimental grouping and exercise plan.

Four altitudes and three exercise modes were tested in combination, yielding a total of 12 groups. Rats in the 410 m group were reared under normoxic conditions (Xizang Minzu University, Weicheng District, Xianyang, Shaanxi Province), and rats in the high‐altitude group were raised in a low‐pressure environment simulation chamber (Xi'an Fukang Air Purification Equipment and Engineering Co., Ltd, Xi'an, China). The details of the groups and exercise plan are shown in Figure 1.

### 2.4 Echocardiography

The whole exercise plan lasted 30 days. Immediately after the last exercise, the rats were anaesthetized with isoflurane inhalation. The rats were first anaesthetized with 3.0–5.0% isoflurane in the induction, and then with 1.0–2.0% isoflurane to maintain the sedation level. The heart rate (HR), stroke volume (SV), left ventricular end‐diastolic volume (LVEDV) and ejection fraction (EF) were measured by a colour ultrasound diagnostic instrument (VINNO Technology Co., Ltd, Suzhou, China) to analyse cardiac function.

### 2.5 Red blood cells and haemoglobin counts

After echocardiography, the inner canthus venous blood was taken for detection of red blood cells (RBCs) and haemoglobin (HGB) with a Mindray BC‐2800 Vet Hematology analyser (Shenzhen, China).

### 2.6 Biochemical detection

After echocardiography, blood samples were collected from the abdominal aorta and then centrifuged at 973 *g* for 10 min to acquire serum for biochemical detection with an enzyme‐linked immunosorbent assay (ELISA) kit (Shanghai Enzyme‐linked Biotechnology Co., Ltd, Shanghai, China). The biomarkers tested were creatine kinase (CK), myoglobin (MB) and cardiac troponin I (cTnI).

### 2.7 Histological staining and analysis

The animals were perfused with 0.1 M ice‐cold phosphate‐buffered saline followed by 4% paraformaldehyde. The hearts of the rats were then postfixed in the same fixative for 48–72 h, embedded in paraffin, and cut into 5‐µm‐thick sections for haematoxylin–eosin (HE) staining to evaluate the degree of myocardial injury. The primary observation indicators include inflammatory cell infiltration, vascular congestion, fibrous tissue hyperplasia and degree of degeneration.

### 2.8 Statistical analyses

The experimental data are expressed as the mean ± standard error. IBM SPSS Statistics 26 (IBM Corp., Armonk, NY, USA) statistical software was used for statistical analysis, and GraphPad Prism 7 software (GraphPad Software Inc., San Diego, CA, USA) was used for plotting. One‐way ANOVA was used for statistical analysis. The final results were statistically significant when *P* < 0.05.

## 3 RESULTS

### 3.1 The variance of red blood cells and hemoglobin

**FIGURE 2:**
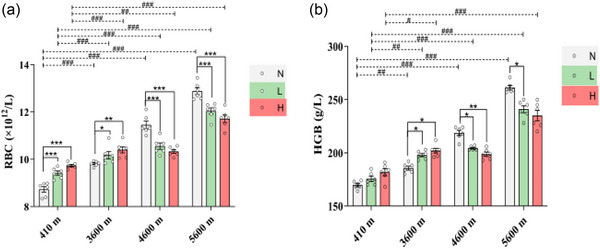
Comparison of red blood cells (RBCs; a) and haemoglobin (HGB; b). Data were analysed by one‐way ANOVA followed by the least significant difference (LSD) test (a) and the Games–Howell post‐hoc multiple comparisons test (b), and are expressed as means ± SEM. *P < 0.05, **P < 0.01 and ***P < 0.001 comparing exercise intensities at the same altitude; #*P* < 0.05, ##*P* < 0.01 and ###*P* < 0.001 comparing altitude conditions at the same exercise intensities.

As shown in Figure 2, for the non‐exercised condition, compared with the 410 N group (RBCs: 8.73 ± 0.12; HGB: 169.67 ± 1.52), the RBCs and HGB of the 3600 N (9.82 ± 0.04, *P* < 0.001; 185.67 ± 1.82, *P* = 0.0018 (*P *< 0.01)), 4600 N (11.46 ± 0.17, *P* < 0.001; 218.67 ± 2.51, *P* < 0.001), and 5600 N (12.88 ± 0.14, *P* < 0.001; 261.33 ± 2.11, *P* < 0.001) groups increased significantly.

For low‐intensity exercise, RBC levels and HGB significantly increased in the 3600 L (10.18 ± 0.16, *P* < 0.001; 197.83 ± 1.60, *P *= 0.0011 (*P *< 0.01)), 4600 L (10.56 ± 0.14, *P* < 0.001; 204.33 ± 0.71, *P* = 0.0006 (*P *< 0.001)) and 5600 L (12.05 ± 0.13, *P* < 0.001; 241.00 ± 3.28, *P* < 0.001) groups relative to the 410 L group (RBCs: 9.42 ± 0.09; HGB:175.50 ± 2.43).

For high‐intensity exercise, compared with the 410 H group (RBCs: 9.73 ± 0.05; HGB: 181.83 ± 3.24), RBCs in the 3600 H (10.40 ± 0.13, *P* < 0.001), 4600 H (10.33 ± 0.08, *P* = 0.0012 (*P *< 0.01)) and 5600 H (11.72 ± 0.16, *P* < 0.001) groups increased significantly, and HGB in the 3600 H (202.00 ± 2.34, *P* = 0.017 (*P *< 0.05)) and 5600 H (235.00 ± 4.89, *P* < 0.001) groups also increased significantly.

### 3.2 Changes in cardiac function

#### 3.2.1 Stroke volume

**FIGURE 3:**
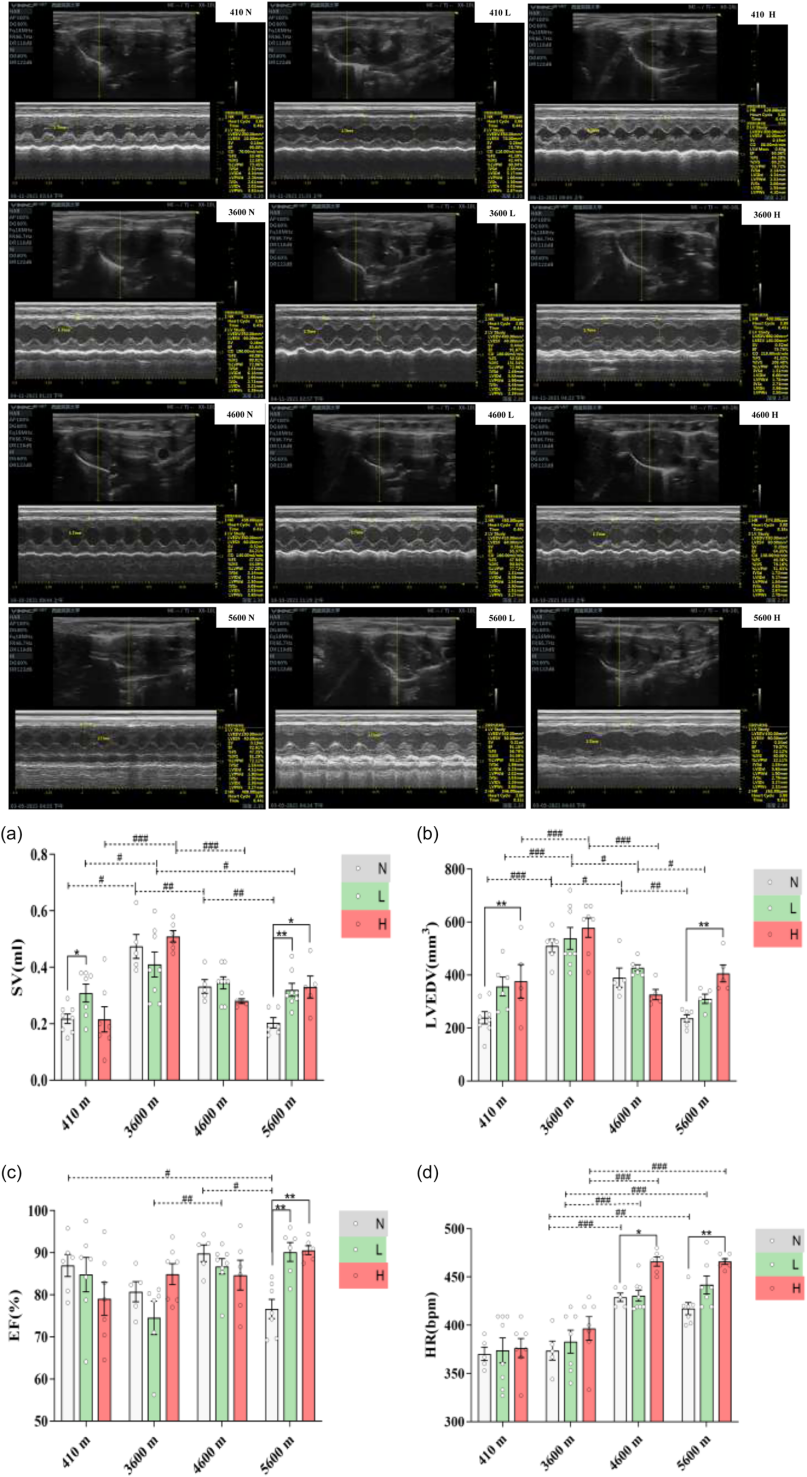
Echocardiograms and statistical analysis. (a) Stroke volume. (b) Left ventricular end‐diastolic volume. (c) Ejection fraction. (d) Heart rate. Data were analysed by one‐way ANOVA followed by the LSD test (c) or the Games–Howell multiple comparisons test (a, b, d) and are expressed as means ± SEM. *P < 0.05 and **P < 0.01 comparing exercise intensities in same altitude; #*P* < 0.05, ##*P* < 0.01 and ###*P* < 0.001 comparing altitude conditions in same exercise intensities.

As shown in Figure 3a, for the non‐exercised condition, the SV in the 3600 N group (0.47 ± 0.05 mL) was higher than that in the 410 N group (0.22 ± 0.02 mL, *P* = 0.0327 (*P *< 0.05)). The SV was lower in the 4600 N group (0.33 ± 0.02 mL, *P *= 0.0057 (*P *< 0.01)) than in the 3600 N group, while the SV was lower in the 5600 N group (0.20 ± 0.02 mL, *P* = 0.0086 (*P *< 0.01)) than in the 4600 N group.

For low‐intensity exercise, the 3600 L group (0.41 ± 0.04 mL) had a greater SV than the 410 L group (0.31 ± 0.03 mL, *P *= 0.0150 (*P *< 0.05)). Compared with the SV of the 3600 L group, the SV of the 5600 L group was lower (0.32 ± 0.02 mL, *P* = 0.0250 (*P *< 0.05)).

For high‐intensity exercise, the 3600 H group (0.51 ± 0.02 mL) had a greater SV than the 410 H group (0.22 ± 0.04 mL, *P* < 0.001), as well as higher SV than the 4600 H group (0.28 ± 0.01 mL, *P* < 0.001).

#### 3.2.2 Left ventricular end‐diastolic volume

As shown in Figure 3b, for the non‐exercised condition, LVEDV was significantly greater in the 3600 N group (510.28 ± 24.63 mm^3^) than in the 410 N group (238.75 ± 22.87 mm^3^, *P *< 0.001). Above 3600 m, LVEDV significantly decreased with increasing altitude (3600 N 510.28 ± 24.63 mm^3^ vs. 4600 N 390.00 ± 36.47 mm^3^, *P* = 0.0165 (*P *< 0.05); 4600 N 390.00 ± 36.47 mm^3^ vs. 5600 N 236.67 ± 13.08 mm^3^, *P* = 0.0026 (*P *< 0.01)).

For low‐intensity exercise, the LVEDV in the 3600 L group (538.33 ± 41.26 mm^3^) was also greater than that in the 410 L group (356.67 ± 35.75 mm^3^, *P* < 0.001). Compared with that in the 3600 L group, the LVEDV in the 4600 L group (426.67 ± 12.56 mm^3^, *P* = 0.0127 < 0.05) was lower, and compared with that in the 4600 L group, the LVEDV in the 5600 L group (310.00 ± 17.61 mm^3^, *P *= 0.0198 (*P *< 0.05)) was even lower.

For high‐intensity exercise, LVEDV was also greater in the 3600 H group (578.57 ± 36.22 mm^3^) than in the 410 H group (376.00 ± 63.92 mm^3^, *P* < 0.001). Compared with that in the 3600 H group, the LVEDV in the 4600 H group was significantly lower (326.00 ± 19.65 mm^3^, *P* < 0.001).

#### 3.2.3 Ejection fraction

As shown in Figure 3c, for the altitude of 5600 m, the 5600 L (90.20 ± 2.26%, *P* = 0.0015 (*P *< 0.01)) and 5600 H (90.60 ± 1.04%, *P* = 0.0011 (*P *< 0.01)) groups had higher EFs than the 5600 N group (76.66 ± 2.30%).

For the non‐exercised condition, the EF of the 5600 N group was lower than that of the 410 N group (86.97 ± 2.63%, *P* = 0.0141 (*P *<  0.05)). Compared with the 4600 N group (89.86 ± 1.98%), the 5600 N group had a lower EF (*P *= 0.0365 (*P *< 0.05)).

#### 3.2.4 Heart rate

As shown in Figure 3d, for the same altitude, the HR of the 4600 H group (466.00 ± 4.67 bpm) was higher than that of the 4600 N group (429.00 ± 4.47 bpm, *P* = 0.0118 (*P *< 0.05)), and the HR of the rats in the 5600 H group (466.00 ± 2.97 bpm) was higher than that of the rats in the 5600 N group (417.29 ± 6.35 bpm, *P* = 0.0025 (*P *< 0.01)).

For the same exercise intensities at different altitudes, the results showed the same trend, as follows: for the non‐exercised condition, compared with those in the 3600 N group (373.80 ± 9.86 bpm), the HRs in the 4600 N group (*P* < 0.001) and the 5600 N group (*P* = 0.0025 (*P *< 0.01)) were greater.

For low‐intensity exercise, the 4600 L group (430.50 ± 5.57 bpm, *P* < 0.001) and the 5600 L group (441.86 ± 9.23 bpm, *P *< 0.001) had faster HRs than the 3600 L group (383.00 ± 11.94 bpm).

For high‐intensity exercise, the 4600 H group (*P* < 0.001) and 5600 H group (*P* < 0.001) had higher HRs than the 3600 H group (396.71 ± 12.25 bpm).

### 3.3 The variance of biomarkers for myocardial injury

#### 3.3.1 CK concentration

**FIGURE 4:**
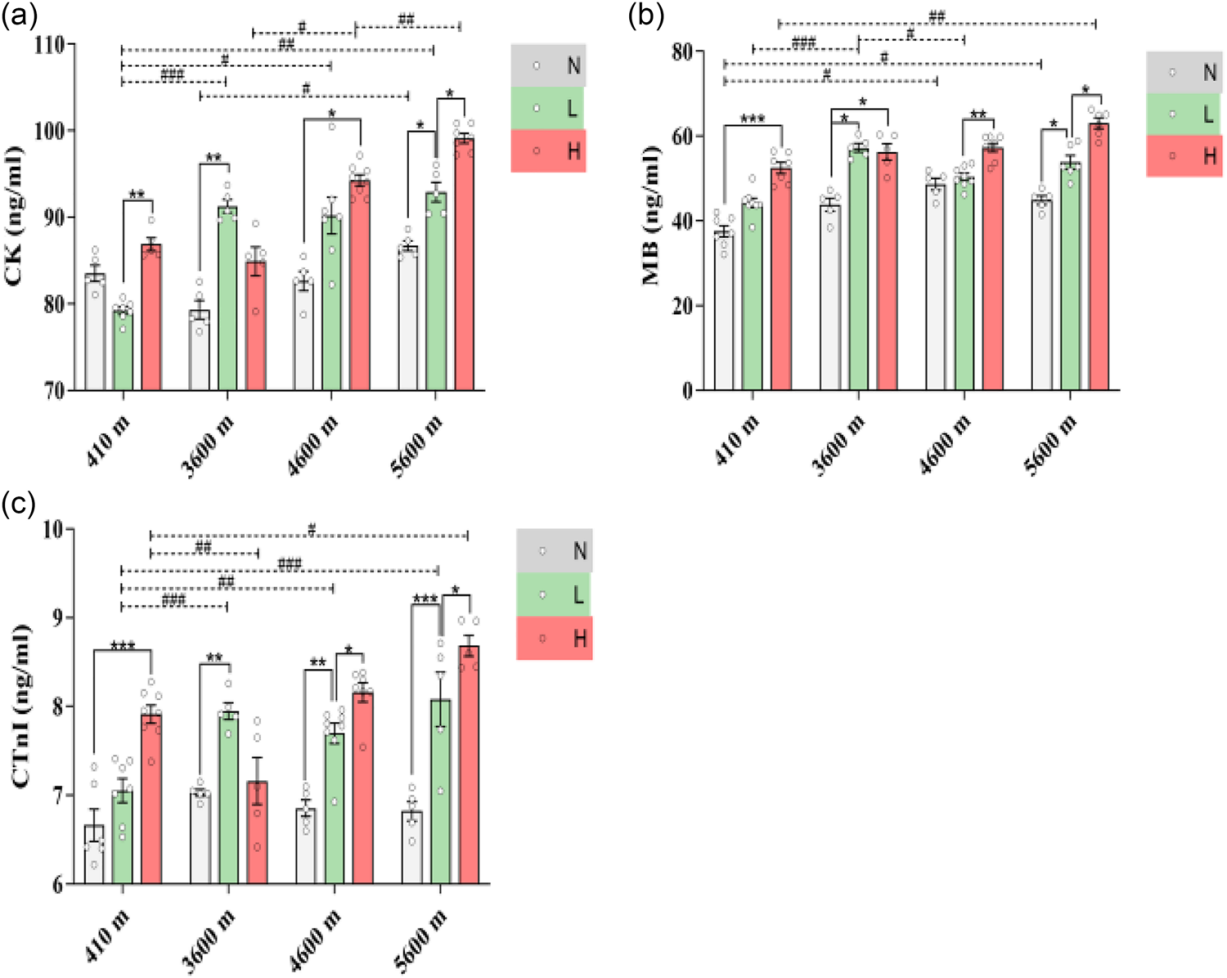
The effects of different altitudes and different exercise intensities on the secretion of myocardial injury markers in rats. (a) Creatine kinase. (b) Myoglobin. (c) Cardiac troponin I. Data were analysed by one‐way ANOVA followed by the LSD test (a, b) or the Games–Howell multiple comparisons test (c) and are expressed as means ± SEM. *P < 0.05, **P < 0.01 and ***P < 0.001 comparing exercise intensities in same altitude; #*P* < 0.05, ##*P* < 0.01 and ###*P* < 0.001 comparing altitude conditions in same exercise intensities.

As shown in Figure 4a, for the non‐exercised condition, the myocardial CK concentration in the 5600 N group (86.70 ± 0.59 ng/mL) was higher than that in the 3600 N group (79.31 ± 1.05 ng/mL, *P* = 0.0143 (*P *< 0.05)).

For low‐intensity exercise, compared with those in the 410 L group (79.23 ± 0.44 ng/mL), the myocardial concentrations of CK in the 3600 L (91.28 ± 0.76 ng/mL, *P* < 0.001), 4600 L (90.21 ± 2.12 ng/mL, *P* = 0.0334 (*P *< 0.05)) and 5600 L (92.91 ± 1.15 ng/mL, *P* = 0.0014 (*P *< 0.01)) groups increased.

Compared with that in the 3600 H group (84.90 ± 1.64 ng/mL), the concentration of CK in the 4600 H (94.29 ± 0.63 ng/mL, *P* = 0.0411 (*P *< 0.05)) group was significantly greater; in turn, it was higher in the 5600 H group (99.13 ± 0.56 ng/mL, *P* = 0.0026 (*P *< 0.01)) than in the 4600 H group.

#### 3.3.2 MB concentration

As shown in Figure 4b, for the non‐exercised condition, there was no significant difference in the MB concentration between the 410 N group (37.55 ± 1.35 ng/mL) and the 3600 N group (43.84 ± 1.55 ng/mL, *P* = 0.2530 (>0.05)); however, the MB concentrations in the 4600 N (48.67 ± 1.40 ng/mL, *P* = 0.0159 (*P *< 0.05)) and 5600 N groups (44.89 ± 1.06 ng/mL, *P *= 0.0408 (*P *< 0.05)) were greater than those in the 410 N group.

For low‐intensity exercise, compared with that in the 410 L group (44.29 ± 1.11 ng/mL), the concentration of MB in the 3600 L group (57.25 ± 1.03 ng/mL, *P* < 0.001) was significantly greater.

For high‐intensity exercise, compared with that in the 410 H group (52.48 ± 1.33 ng/mL), the concentration of MB in the 5600 H group increased (63.03 ± 1.24 ng/mL, *P* = 0.0038 (*P *< 0.01)).

#### 3.3.3 cTnI concentration

As shown in Figure 4c, for low‐intensity exercise, the cTnI concentrations were greater in the 3600 L (7.95 ± 0.09 ng/mL, *P *< 0.001), 4600 L (7.70 ± 0.12 ng/mL, *P *= 0.0010 (*P *< 0.01)) and 5600 L (8.08 ± 0.31 ng/mL, *P* < 0.001) groups than in the 410 L group (7.05 ± 0.13 ng/mL).

For high‐intensity exercise, compared with that in the 410 H group (7.91 ± 0.10 ng/mL), the concentration of cTnI in the 5600 H group (8.68 ± 0.12 ng/mL, *P* = 0.0193 (*P *< 0.05)) was greater.

### 3.4 HE staining of the heart

**FIGURE 5:**
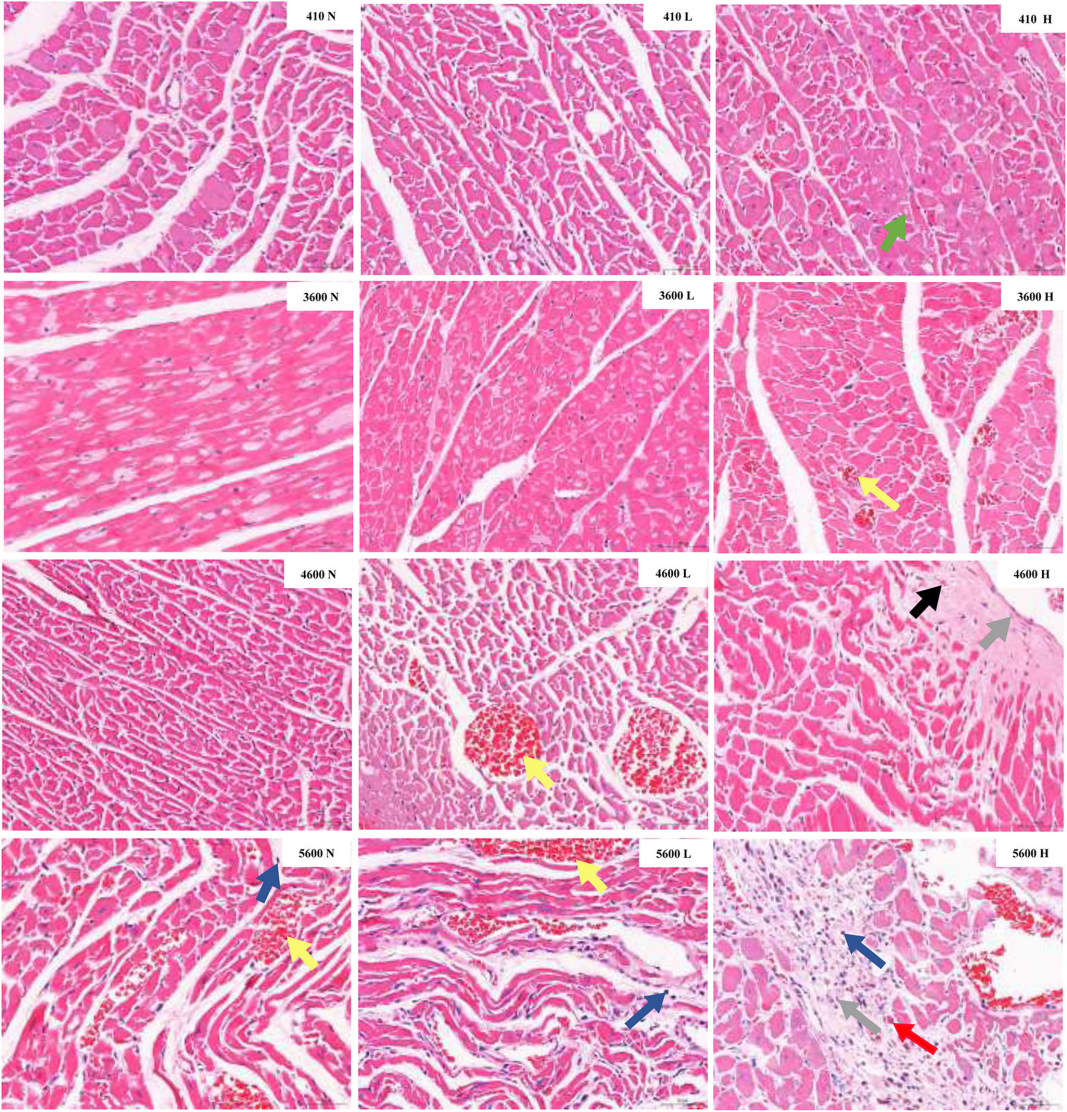
HE staining of rat hearts at different altitudes and different exercise intensities (HE ×400). Myocardial fibre vacuolar degeneration (↑), vascular congestion (↑), lymphocytes (↑), fibrocyte (↑), new capillaries (↑) and myocardial fibre necrosis (↑). Scale bar, 50 μm.

After 4 weeks of the experiment, HE staining was used to observe the morphological changes in the heart. The results showed that (a) for the non‐exercised condition, inflammatory cell infiltration, slight vascular congestion, and a small amount of fibrous tissue hyperplasia were observed in the myocardial interstitium of the 5600 N group, while no significant pathological changes were observed in the 410 N group, 3600 N group or 4600 N group. (b) For low‐intensity exercise, a small amount of vacuolar deformation and vascular congestion were observed in the 4600 L group. In the 5600 L group, some blood vessels were slightly congested, and some lymphocyte infiltration was observed in the interstitium. There was no obvious abnormality in the 410 L or 3600 L group. (c) For high‐intensity exercise, vacuolar degeneration of cardiomyocytes appeared in the 410 H group. In the 3600 H group, vascular congestion was observed. In the 4600 H group, vacuolar degeneration or even necrosis in a few myocardial fibres was observed, as well as some fibrous tissue hyperplasia and vascular congestion in the necrotic area. In the 5600 H group, there was a small amount of myocardial fibre necrosis, with inflammatory cell infiltration and fibrous hyperplasia; meanwhile, a small amount of new capillary formation and vascular congestion were visible (Figure 5).

## 4 DISCUSSION

Hypoxia disrupts energy metabolism and promotes the accumulation of oxygen free radicals in cardiomyocytes, which increase the permeability of the cell membrane, thereby causing the release of myocardial enzymes into cells and inducing myocardial injury (He et al., 2021).

Cardiac function indices are vital for evaluating an individual's health status and physical activity capabilities. The present experiment revealed that, for the same exercise intensity, the SV and LVEDV were the highest in the 3600 m group, and this group's HR and EF were similar to those in the 410 m group. However, as altitude increased to 4600 and 5600 m, notable decreases in SV and LVEDV were observed, accompanied by an accelerated HR. These results underscore that, at equivalent exercise intensities, an altitude of 3600 m enhances cardiac pumping. Conversely, the decline in SV and LVEDV at 4600 and 5600 m is attributed to the combined influence of high altitude exposure and exercise intervention. As the heart encounters greater challenges in pumping blood efficiently, it compensates by elevating its beat rate to maintain overall cardiac function.

Serum levels of CK, MB and cTnI are widely recognized markers for diagnosing acute myocardial injury (Antman, 2018; Chen et al., 2022; Seto, 2019). When myocardial injury factor levels and pathological results were integrated, the cardiac function of rats in the non‐exercised state at 3600 m was significantly improved without notable secretion of injury factors or myocardial pathological damage. Conversely, at altitudes of 4600 and 5600 m, cardiac function declined, accompanied by elevated injury marker levels and myocardial injury. The results suggested that the best adaptation altitude of the heart may be near 3600 m. Notably, the majority of Tibetans (85%) reside at an altitude ranging from 2500 to 4500 m, with only 6.2% inhabiting above 4500 m. Higher altitudes (>4500 m) will pose a potential challenge to their lives and health (Stembridge et al., 2016). Zhang et al. (2017) identified an inflection point at approximately 4500 m in Tibet, where the concentration of haemoglobin and polycythaemia rates undergo a sharp increase. Our research further corroborates this finding, revealing that both red blood cell counts and haemoglobin levels exhibit a notable elevation as altitude increases, with the most pronounced variation observed at an altitude of 4600 m. Of course, organs and systems exhibit optimal altitudes for hypoxic adaptation, due to varying structures, functions and roles. The present study indicates that the heart's optimal altitude for adaptation may lie close to 3600 m.

Beyond the effect of altitude, the influence of exercise on the heart ought not to be overlooked. Exercise is instrumental in mitigating oxidative stress and minimizing myocardial injury, thereby making it a pivotal approach not merely for preventing pathological cardiac remodelling but also for improving cardiac function (Ma et al., 2021; Marino et al., 2021). Nevertheless, engaging in excessive exercise markedly escalates the production of free radicals within cardiomyocytes, triggering the release of myocardial enzymes (Gao et al., 2021). Notably, the 3600 m group exhibited no significant differences in myocardial enzyme levels or pathological myocardial damage compared to the 410 m group. Conversely, at altitudes of 4600 and 5600 m, an upward trend in myocardial enzyme concentrations and pathological injuries to myocardial cells was observed, correlated with increasing exercise intensities.

These findings indicate a positive correlation between the severity of myocardial injury and both altitude and exercise intensity. It is generally believed that traditional or classic altitude training, residing and training at moderate altitudes (around 1500–3000 m), enhances sea‐level performance (Carr et al., 2015; Saunders et al., 2019). This aligns with our findings that exercise at 3600 m or below can enhance cardiac function, thereby improving sports performance. For individuals residing at middle and high altitudes, it is imperative to adjust exercise intensity downwards as altitude increases. Furthermore, the development of individualized, scientific and adaptable exercise regimens tailored to specific needs becomes paramount.

### 4.1 Conclusions

(a) This paper's experimental design indicates 3600 m as a pivotal threshold where cardiac function begins to decline, potentially triggering subtle myocardial inflammation even at rest. (b) The alterations in cell secretion, tissue architecture and organ function occur asynchronously yet are interconnected. Notably, in this experiment, myocardial cell secretion levels shifted initially, hinting that for early detection of myocardial injury at high altitudes, focusing on specific molecular markers may surpass cardiac structural assessments. For early warning of myocardial injury in non‐exercise states at extreme altitudes, particular attention should be paid to alterations in myocardial MB levels during molecular indicator detection. (c) EF, the SV/LVEDV ratio, remains stable across groups due to isotropic changes of SV and LVEDV. Holistic evaluation of cardiac function, considering multiple indicators, is crucial. Despite limitations in recording exercise HR, resting findings show exercise's enduring effects on cardiac function. This study has deepened our understanding of the effects of plateau exercise on the heart and provided new insights into the exploration and formulation of appropriate plateau exercises.

## AUTHOR CONTRIBUTIONS

Minxia Zhu: Conceptualization, data curation, funding acquisition, project administration, supervision, and writing—review and editing. X. Li and Bin Liu: Formal analysis, methodology, and writing—original draft. Jing Guo: Data curation and writing—review and editing. Yuanyuan Xiao, Zhiyao Liu, Mengru Duan, and Yi Liu: Methodology and performed experiments. All authors have read and approved the final version of this manuscript and agree to be accountable for all aspects of the work in ensuring that questions related to the accuracy or integrity of any part of the work are appropriately investigated and resolved. All persons designated as authors qualify for authorship, and all those who qualify for authorship are listed. No generative AI and AI‐assisted technologies in the writing process has been used.

## CONFLICT OF INTEREST

The authors declare no conflicts of interest.

## Data Availability

The data that support the findings of this study are available from the corresponding author upon reasonable request.
